# Reproductive adaptations of the hydrothermal vent crab *Xenograpus testudinatus*: An isotopic approach

**DOI:** 10.1371/journal.pone.0211516

**Published:** 2019-02-07

**Authors:** Jia-Jang Hung, Shao-Hung Peng, Chen-Tung Arthur Chen, Tsui-Ping Wei, Jiang-Shiou Hwang

**Affiliations:** 1 Department of Oceanography, Asian-Pacific Ocean Research Center, National Sun Yat-sen University, Kaohsiung, Taiwan; 2 Center for Research in Water Science & Technology, National Cheng Kung University, Tainan, Taiwan; 3 Institute of Fisheries Science, National Taiwan University, Taipei, Taiwan; 4 Institute of Marine Biology, National Taiwan Ocean University, Keelung, Taiwan; 5 Center of Excellence for the Oceans, National Taiwan Ocean University, Keelung, Taiwan; Kaohsiung Medical University, TAIWAN

## Abstract

The vent crab *Xenograpsus testudinatus* was firstly discovered in 2000 at the hydrothermal vent field off the coast of Kueishan Island. The present study attempts to understand the adaptive reproduction of this crab living in an extreme environment by examining its spatial and temporal distribution and isotopic signatures. The seasonal variation of the female-male ratio suggests that ovigerous females may migrate from beneath the vent orifice to the vent-periphery region to release their larvae to avoid the larvae contacting high toxic plumes, and then returns to the vent orifice habitat. We used variation of the isotopic crab signatures as indicators for this unique female migration. Our results showed that this vent crab evolved an adaptive modulation of reproductive behavior to successfully survive and propagate in an oceanic shallow hydrothermal vent field.

## Introduction

Hydrothermal vents exist in locations ranging from coastal ridges to the abyss. Light is most likely absent at depths beyond 200 meters, and biological communities are dominated by numerous symbiotrophic forms, sustained by chemosynthesis using sulfide compounds as their primary energy source [1, 2]. In contrast, shallow hydrothermal vents may erupt within the euphotic zone and the species living around them are not obligate taxa; few organisms exist near these shallow vents [2, 3].

Kueishan Island, a young active volcanic island, is located approximately 10 km off the northeastern coast of Taiwan. To date, the shallow hydrothermal vents found near Kueishan Island have predominantly been found on its eastern side. *Xenograpsus testudinatus* is currently the only crustacean species known to live in the shallow hydrothermal vent field of Kueishan Island. This vent crab is believed to have lived in the area for a long time, but was only discovered in 2000, and appears to have adapted to such extreme local environment. Jeng *et al*. [4] proposed that *X*. *testudinatus* living in the vent field likely fed on zooplanktons that were killed by the vent plumes and then settled on the sea-bed like ‘snow.’ Although some limited reports have examined the diet, metabolic and behavioral adaptions of *X*. *testudinatus* in the shallow hydrothermal vents [5, 6, 7] reproductive adaptation mechanisms are unknown as yet.

In deep hydrothermal vents, the ovigerous female of the vent crab *Bythograea thermydron* has only been observed in the vent periphery and not in the vent orifice [8]. Therefore, it would be interesting to understand the reproductive adaptations and possible seasonal migrations for the shallow hydrothermal vent crab *X*. *testudinatus*. This study attempts to elucidate crab adaptation and reproductive biology in this extreme environment by examining crab ovigerous rates, seasonal shifts in sex ratio, isotopic signatures and possible food sources.

## Materials and methods

**Fig 1:**
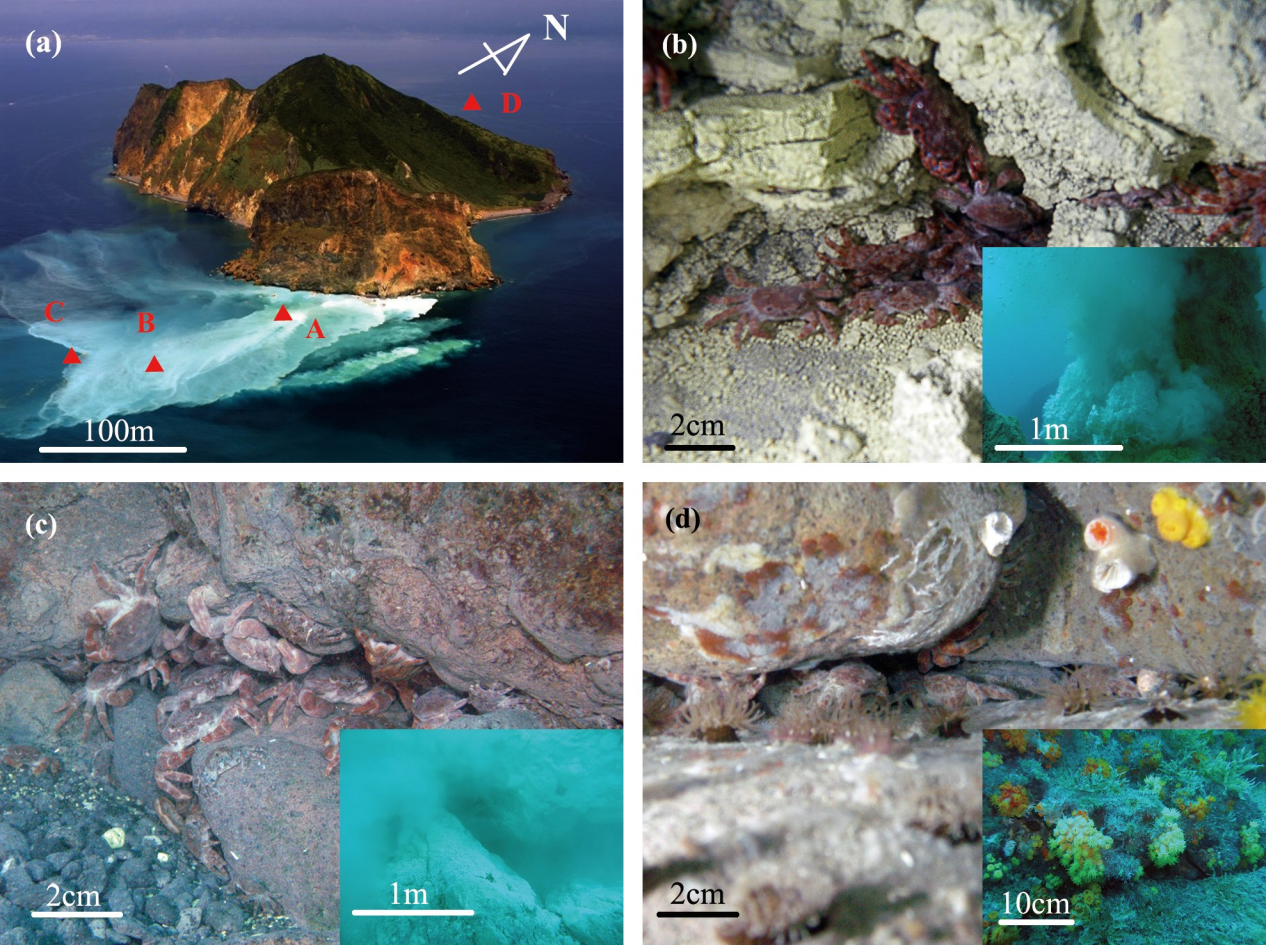
(a) Sampling sites around Kueishan Island. Sites ‘A,’ ‘B’ and ‘C’ are in the vent field and site ‘D’ is in the coral reef area. (b) Underwater view of site ‘A’, characterized by a yellow vent with a chimney, as shown in the inset. The crabs live around the base of the vent containing sulfide-enriched sediment, an environment free of other organisms. (c) Site ‘B’ has a white vent crevice without chimney, as shown in the inset. The crabs aggregate in rock crevices inhabited by anemones and algae. (d) Site ‘C' is located on the edge of the vent field. Crabs aggregate in rock crevices along with other organisms like algae, anemone, coral, snails, etc. The biological community resembles that in coral reef areas.

In this study we analyzed seawater, sediment, and biological samples collected by researchers through three independent samplings from shallow water hydrothermal vents, conducted in April 2010, July 2010, and July 2011 at four sites (**Fig 1**). The crab *Xenograpsus testudinatus* and linked organisms were caught in April and July 2010. Crabs were not caught at site ‘B’ in the April sample because of extremely bad weather. Site ‘A’, with a vent orifice, is located on the eastern side of Kueishan Island. Site ‘B’ is also a vent location, emitting fluids likely from crevices, but located some distance from site ‘A.' Meanwhile, site ‘C’ is located on the edge of the vent field where *X*. *testudinatus* can be found with other organisms. Finally, site ‘D’ is a coral reef area located in the north of Kueishan Island where no vent crabs can be found. Additionally, a home-made sediment trap (**S1 Fig**) was deployed in both vent field and coral reef sites for eight days during September 2011 to collect settling materials.

During crab and sediment sampling expeditions, temperature, dissolved oxygen (DO), pH, and total dissolved sulfide (TDS) in seawater were measured *in situ* or in the laboratory. Total organic carbon (TOC) and nitrogen (TN), and total inorganic carbon (TIC) and inorganic sulfur (TS) in sediment and trap samples were measured according to the previous methods of Hung et al. [9, 10, 11]. The food web structure was examined using stable isotopic (δ^13^C and δ^15^N) data measured from *X*. *testudinatus* and other organisms caught in July 2010 according to the analytical method of Liu et al. [12]. The collected crabs were carefully examined to determine their carapace width, weight, sex, and ovigerous status.

The data of δ^13^C and δ^15^N in *X*. *testudinatus*, ovigerous rate and elemental composition in sediment from each sampling site were statistically analyzed using ANOVA and then compared using the Fisher LSD test. The student t-test was used to compare the differences in elemental composition between the trap samples collected from the vent field and coral reef field of female and ovigerous females.

## Results

**Fig 2:**
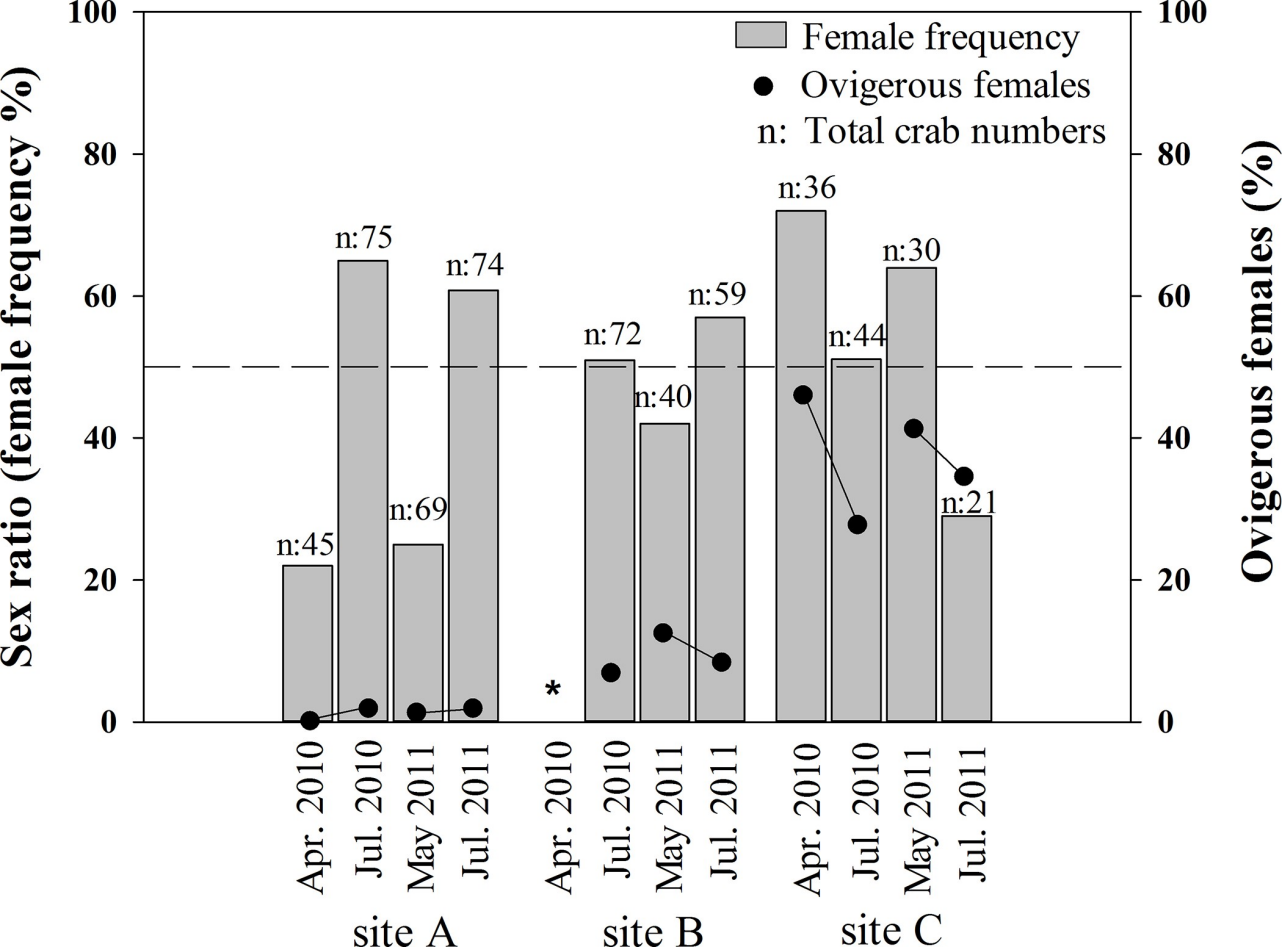
Ovigerous ratios of *X*. *testudinatus* females collected from three sites. Distribution of isotopic signatures of carbon and nitrogen in organisms collected from the Kueishan hydrothermal field. The geometric marks indicate (mean ± SD) in *X*. *testudinatus* samples. Open: male; solid: female; triangle: samples from site ‘A’; square: samples from site ‘B’; circle: samples from site ‘C’.

**Fig 3:**
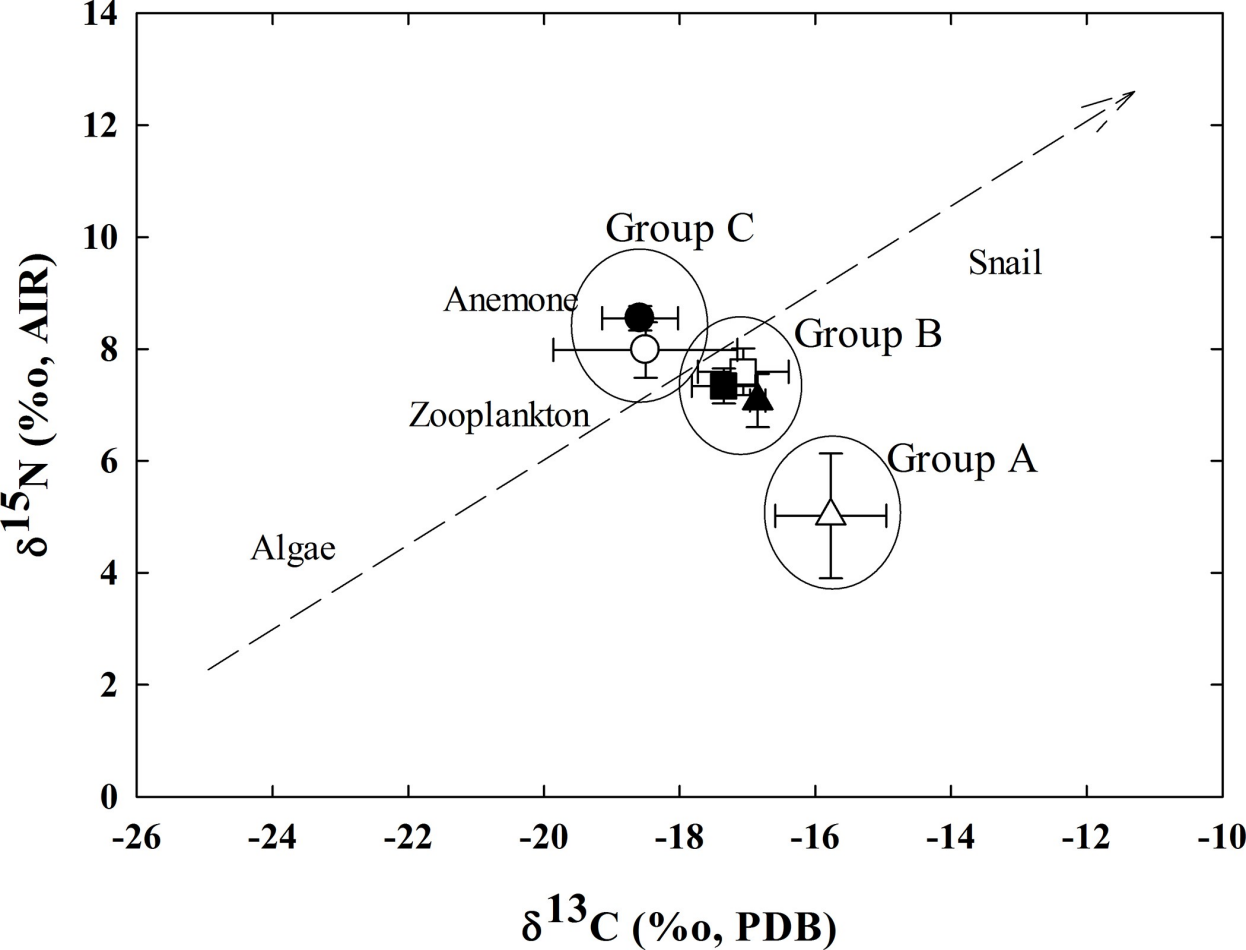
Distribution of isotopic signatures of carbon and nitrogen in organisms collected from the Kueishan hydrothermal field. The geometric marks indicate (mean ± SD) in *X*. *testudinatus* samples. Open: male; solid: female; triangle: samples from site ‘A’; square: samples from site ‘B’; circle: samples from site ‘C’.

Comparing the female ratio and ovigerous female ratio of the vent crabs at the three collection sites during different seasons (**Fig 2**) demonstrated that the crabs from beneath the vent orifice (site ‘A’) have total female and ovigerous female ratios of 22% and 0% in April, respectively. The total female and ovigerous female ratios increased to 65% and 2.04% in July 2010. In contrast, the total female and ovigerous female ratios at site ‘C’ in April were 72% and 46.15%, respectively. Both ratios decreased to 48% and 28.87%, respectively, in July 2010. The results indicate that spring is the main reproductive season of *X*. *testudinatus*, and the mature female crab may exhibit unique seasonal-migration behavior during this period. The isotopic signatures of collected female and male crabs also indicated the unique migratory behavior of female crabs (**Fig 3**).

In this study, δ^13^C and δ^15^N data of different organisms collected from the Kueishan Island (**Fig 3**) suggested that the food web structures contained primary and secondary consumers. The isotopic signatures of male crabs collected during July 2010 were categorized into three different groups (group ‘A’, ‘B’, and ‘C’), corresponding to their different habitats (site ‘A’, ‘B’ and ‘C’) (**Fig 2**). The isotopic signatures in group ‘A’ differed significantly from those in groups ‘B’ and ‘C’ (p < 0.01, both in δ_13_C and δ^15^N). However, the signatures in group ‘B‘ resembled those in group ‘C’.

The vent fluid collected at site ‘A’ showed high temperature (106°C), low pH (2.48), low DO (47.3 μM), and high TDS (26.5 mg L^-1^). The seawater samples collected from the bottom of site ‘A’ (the bottom of the vent chimney) showed values similar to the ambient ocean waters: temperature (27°C) and DO (179.5 μM). These waters show a slightly lower than normal pH (6.38) and TDS (5.49 mg L^-1^). Such distributions do not differ significantly compared to those from the non-vent region (coral reefs, site ‘D’), except in pH and TDS (**Table 1**). Comparing the composition of the settling materials collected from vent and coral reef fields revealed significantly higher concentrations of total organic carbon (TOC) and total sulfur (TS) in the vent field (TOC: 4.23 mg g^-1^; TS: 23.54%) than the coral reef field (TOC: 3.05 mg g^-1^; TS: 0.88%) (**Table 2**). The low C/N ratios detected in the settling materials from the vent field may also imply that they have a relatively high zooplankton content, which was confirmed by examination using a microscope (**Fig 2**).

**Table 1:** Distributions of temperature, pH, DO and TDS in vent fluids and ambient sea water near Kueishan Island.

Sites	Depth (m)	Class	Temperature (°C)			pH			Total dissolved sulfide (mg L^-1^)			Dissolved oxygen (μM)		
A	10	Vent fluid	106	±	6	2.48	±	1.06	26.54	±	21.92	47.3	±	37.8
B	17	Vent fluid	55	±	4	5.45	±	0.65	54.10	±	25.67	61.0	±	44.7
A	12	Crab habitat	27	±	2	6.38	±	0.97	5.49	±	5.47	179.5	±	34.2
B	17	Crab habitat	26	±	3	6.86	±	0.86	4.78	±	7.65	192.0	±	26.1
C	19	Crab habitat	25	±	2	7.65	±	0.43	0.58	±	0.54	197.6	±	30.4
D	9	Coral reefs	27	±	2	8.00	±	0.06	ND			218.2	±	11.1

(ND: not detectable)

**Table 2:** Elemental composition in sediment and trap samples collected from each site.

Sites	class	Total sulfur (%)			Total inorganic carbon (mg g^-1^)			Total organic carbon (mg g^-1^)			C/N (mole/mole)		
Vent field	Settling materials	23.54	±	0.26^A^	0.18	±	0.08^A^	4.23	±	0.32^A^	6.44	±	0.36^A^
Coral reefs	Settling materials	0.88	±	0.06^B^	0.28	±	0.03^B^	3.05	±	0.18^B^	7.32	±	0.30^B^
A	Sediment	85.00	±	4.55^a^	0.82	±	0.62^a^	0.86	±	0.51	4.99	±	0.43^a^
B	Sediment	65.20	±	13.79^b^	0.65	±	0.33^a^	0.64	±	0.13	5.90	±	0.51^b^
C	Sediment	22.45	±	9.32^c^	1.04	±	0.41^a^	1.83	±	1.47	6.40	±	0.44^b^
D	Sediment	1.23	±	0.98^d^	3.19	±	1.22^b^	0.80	±	0.62	8.54	±	0.76^c^

The superscripts a, b, c. and d represent the differences in sediment between different sites (ANOVA), while A and B represent the difference in trap samples between the two fields (t-test).

## Discussion

Analysis of stable isotopic signatures of δ^13^C and δ^15^N is a common method used to identify the food web structure of organisms. Since the variation of δ^13^C and δ^15^N isotopic signatures reflects the longer term changes in food sources coming from the same food organism, these signatures were also applied to study the geographic migration or translocation of marine organisms, including crustaceans [13, 14]. δ^13^C and δ^15^N data of different organisms collected from Kueishan Island (**Fig 3**) suggested that the food web structures consisted of primary producers and secondary consumers. For the female crab, the isotopic signatures of specimens collected from site ‘A’ were categorized into groups ‘B’ and ‘C’ but not group ‘A’. Apparently, the non-ovigerous female individuals at site ‘A‘ were recent migrants from sites ‘B’ or ‘C’ in July 2010. This adaptive behavior may be related to the physical, chemical and biological characteristics of their habitats.

Since the vent fluid collected at site ‘A’ showed high temperature (106°C), low pH (2.48), low DO (47.3 M), and high TDS (26.5 mg L^-1^), this fluid is potentially toxic and fatal for crabs. However, the seawater collected from the bottom of site ‘A’ (the bottom of the vent chimney) showed values similar to the ambient ocean waters: temperature (27°C) and DO (179.5 M); these waters show a slightly lower than normal pH (6.38) and TDS (5.49 mg L^-1^). Such distributions do not differ significantly compared to those from the non-vent region (coral reefs, site ‘D’), except in pH and TDS (**Table 1**). This way adult crabs which stay at the bottom avoid direct contact with hot and toxic fluids.

Larvae which exhibit planktotrophic behavior (electronic supplementary material, **S1 Video**) will drift with the upwelled vent fluid and may suffer immediate injury or death if they were released by an ovigerous crab at site ‘A’. Consequently, the mature female crabs migrate to the vent-periphery region (site ‘B’ or ‘C’), about 100–200 meters horizontally away from the vent opening to release their larvae in the ambient environment there, which is more conducive to the larval development.

The reason for female crabs migrating back to their original habitat, the vent field after releasing their larvae is an interesting issue. Lack of competing organisms or predators and rich food sources in the vent field are possible explanations. The settling materials in the coral reef field were characterized by less zooplankton but more macro-algal fragments and mineral particles (**S2 Fig**). This indicated that these materials largely resulted from the resuspension of bottom sediments. In conclusion, this study demonstrated that the vent crab *X*. *testudinatus* has developed a unique reproductive migratory behavior as an adaptation to the shallow hydrothermal vent field. The mature female crabs migrate to the vent-periphery to release their offspring to prevent injury and toxic effects from hot and toxic vent fluids. After larval release they migrate back to the vent field, an area enriched with zooplankton food and with minimal competition or predation from other organisms, indicating a behavioral reproductive adaptation to the vent system. The present study supports the concept of marine hydrothermal vents as peculiar oceanic environments as proposed by Dahms et al. [15], demonstrating here reproductive adaptations of a very particular marine invertebrate to its extreme habitat.

## Supporting information

S1 Fig Deployment of the sediment trap. Left: The structure and setting of the sediment trap. The trap was deployed about 5m above the sea bottom. The length of trap tube is 55cm and has one membrane (diameter: 90mm; pore size: 4μm; polycarbonate) inside to collect the sink materials. Right: The picture of the trap *in situ*.
(DOCX)

S2 Fig The sinking materials collected from two sampling sites. Left: vent field, with many zooplankton (small materials with red color); Right: coral reef field, less zooplankton but have more macro algae fragments than vent field samples.
(DOCX)

S1 Video Larval loss and spawning behavior in the crab vent crab *Xenograpus testudinatus*. (AVI)
