# Supplementary figures and images for: Reproductive adaptations of the hydrothermal vent crab Xenograpus testudinatus: An isotopic approach
Source: PLoS One. 2019 Feb 7;14(2):e0211516. doi: 10.1371/journal.pone.0211516 (PMC6366749; doi:10.1371/journal.pone.0211516)

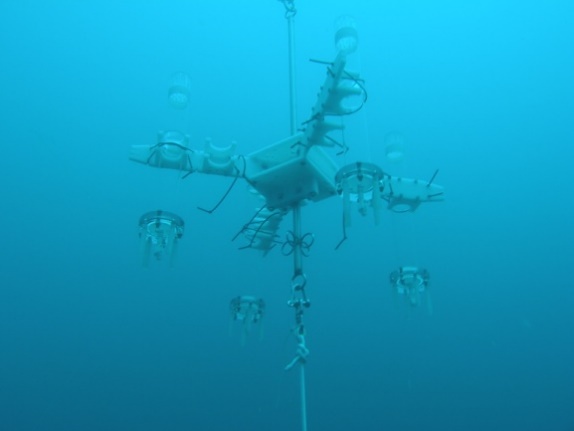

Supplement: S1 Fig — Left: The structure and setting of the sediment trap. The trap was deployed about 5m above the sea bottom. The length of trap tube is 55cm and has one membrane (diameter: 90mm; pore size: 4μm; polycarbonate) inside to collect the sink materials. Right: The picture of the trap in situ. (DOCX) [file pone.0211516.s001.docx]

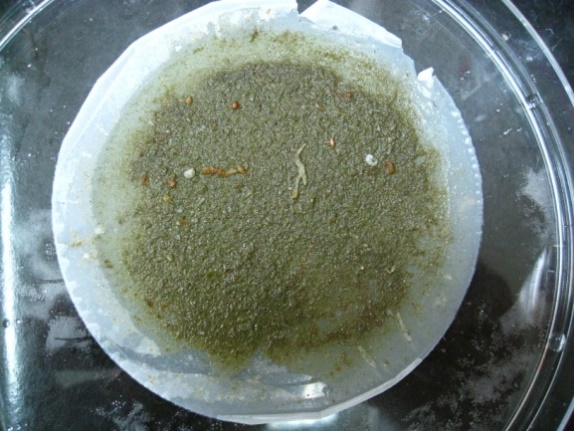

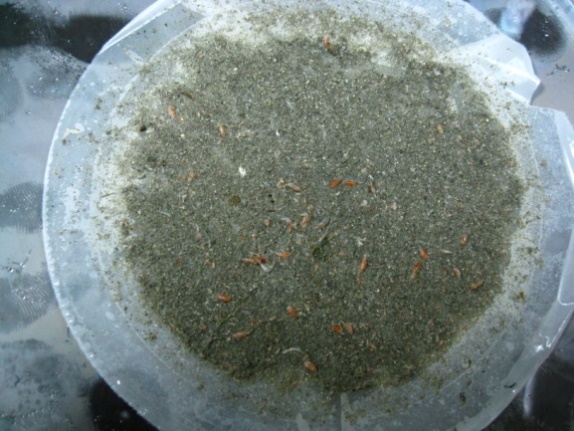

Supplement: S2 Fig — Left: vent field, with many zooplankton (small materials with red color); Right: coral reef field, less zooplankton but have more macro algae fragments than vent field samples. (DOCX) [file pone.0211516.s002.docx]
